# Persistence of Avian Influenza Viruses in Various Artificially Frozen Environmental Water Types

**DOI:** 10.1155/2012/912326

**Published:** 2012-10-04

**Authors:** Dany Shoham, Alam Jahangir, Sakchai Ruenphet, Kazuaki Takehara

**Affiliations:** ^1^Laboratory of Zoonoses, School of Veterinary Medicine, Kitasato University, 35-1 Higashi 23 Bancho, Towada, Aomori 034, Japan; ^2^Animal Health Research Division, Bangladesh Livestock Research Institute, Savar, Dhaka 1341, Bangladesh; ^3^Laboratory of Animal Health, Department of Veterinary Medicine, Tokyo University of Agriculture and Technology, Tokyo 183-8509, Japan

## Abstract

*Background*. This study investigates the viable persistence of avian influenza viruses (AIVs) in various types of artificially frozen environmental water and evaluates the feasibility of similar occurrence taking place in nature, and allowing for prolonged abiotic virus survival, with subsequent biotic viral recirculation. *Methods*. Fresh, brackish, and salty water, taken in Japan from aquatic biotopes regularly visited by migratory waterfowl, were seeded with AIVs. We monthly monitored the viability of the seeded viruses in the frozen state at −20°C and −30°C, for 12 months. We also monitored virus viability following repeatedly induced freezing and thawing. *Results*. The viruses exhibited considerable viable persistence all along that period of time, as well as during freezing-thawing cycles. Appreciable, yet noncrucial variances were observed in relation to some of the parameters examined. *Conclusions*. As typical waterborne pathogens of numerous northerly aquatic birds, AIVs are innately adapted to both the body temperature of their hosts (40°C to 42°C) and, presumably, to subzero temperatures of frozen lakes (down to −54°C in parts of Siberia) occupied and virus-seeded by subclinically infected birds, prior to freezing. Marked cryostability of AIVs appears to be evident. Preservation in environmental ice has significant ecophylogenetic and epidemiological implications, potentially, and could account for various unexplained phenomena.

## 1. Background

A wide diversity of bacteria, protozoa, and viruses are known to exist in various water bodies worldwide, including ponds, lakes, seas, and oceans [1]. In arctic and sub-arctic regions, those water bodies are frozen, entirely or partially, for 4 months (in the southern Taiga) up to 10 months (in the northern Tundra and Arctic Ocean), annually, in the form of seasonal ice. In the Arctic, perennial ice is found too across the Arctic Ocean and freshwater bodies located in Greenland and islands of the Arctic Ocean. All those water bodies are abundantly visited by migratory aquatic birds whenever partially or completely thawed. Consequently, microorganisms that are shed through feces by the birds into water become waterborne, until contracted again by a host or entrapped within refreezing water. In the case of viruses, as obligatory parasites, they are otherwise apt to perish, sooner or later; hence, whenever entrapped in ice, their cryotolerance might constitute a critical factor, in terms of persisting viability, meaning infectivity. The higher their cryotolerance, the longer is the period of time they are liable to viably be preserved in environmental ice, until thawing reoccurs.

Influenza A virus (IAV) is prevalent within some mammals, including man, and multiple avian host species. It is well established that wild birds infected with avian influenza viruses (AIVs)—usually sub-clinically—are infectious for approximately 6–10 days, during which they continuously shed vast concentrations of viral particles in their feces [2, 3]. Large portion of this viral mass is deposited into water by aquatic birds, which are highly permissive of AIVs and frequently infected. A part of the deposited virions are then ingested by susceptible birds occupying the water, thus completing the fecal/oral transmission route [4, 5]. This apparatus is well recognized throughout the Tundra and the Taiga belts. In arctic marine environments, various sea-birds, as well as seals and whales harboring IAVs, cyclically introduce them into local melting-freezing seawater. During spring and summer, sub-clinically AIV-infected migratory aquatic birds are prone to reach any frozen water body across the sub-arctic and arctic regions upon thawing, and seed those water bodies with viruses until freezing reoccurs. During fall, those avian populations are inclined to reach poultry farms, especially in Southeast Asia, thereby allowing for a dynamic viral interface with agricultural- and human-derived influenza strains.

Breban et al. [6] observed that abiotic environmental perpetuation of AIVs ought to regularly take place for periods of 2 years at least, due to offering a parsimonious explanation of the 2–4-year periodicity of avian influenza epidemics; provision of a virus persistence mechanism within small communities where epidemics cannot be sustained by direct transmission only (i.e., communities smaller than the critical community size); and the very low levels of environmental transmission (i.e., few cases per year) that are sufficient for avian influenza to endure within populations where it would otherwise vanish. Year-by-year perpetuation of AIVs in Alaskan lakes occupied during summertime by migratory waterfowl has indeed been pointed at by Ito et al. [7] 

The feasibility of viable preservation of AIVs in lake ice has been proposed by Shoham [8, 9] and Webster et al. [10]. Experimentally, IAVs were indeed found to be fairly cryotolerant, in general [11, 12], as elaborated on below. In addition, occasional unconformities between expected and empirical rates of nucleotide substitutions evidenced within AIVs indicate possible pauses of years without virus replication [13]. At the same time, influenza viruses are not known to and most probably do not undergo dormant phases in their hosts, meaning that there has to be some abiotic mechanism accounting for such genetic conservation. Also, fundamentally, the following open question surfaces anyway: what practically happens to influenza virions—which are known to commonly be found in lake water occupied by waterfowl—when freezing takes place in arctic and subarctic regions, in terms of virus survival? 

On those grounds, the present study assumes, conceptually, what follows.

- Influenza virions can survive in lake water during the relatively short period of time between ice thawing and refreezing, in sub-arctic and arctic regions.
- Biophysically, influenza virions are capable to:
   survive freezing;
   persist viably in the frozen state until thawing occurs next year (or later);
   survive thawing;
   persist again in lake water until recontracted by waterfowl.
- Portions of the virion population survive repeated cycles of freezing and thawing until eventually contracted by waterfowl.

All over, this apparatus may underlie in nature:

 year-to-year viable preservation of viruses that were released from ice upon lake/sea ice thawing but have not been contracted by birds until next freezing occurred, few or several months later;
 multyear preservation of viruses in perennial ice;
 reemergence and recirculation of past strains that as such have advantage over decreasing host herd immunity against them;
 unexplained genetic conservation found from time to time within IAV genomes or genes, in contradiction to the expected genetic drift biomolecular clock (annual rate of nucleotide substitutions).

## 2. Materials and Methods

### 2.1. Water Types

Three types of environmental water, namely, salty, brackish, and fresh water, were collected in Japan as follows:

- fresh water from Lake Izunuma (Miyagi prefecture);
- brackish water from Lake Ogawara (Aomori prefecture);
- salty water from Mutsu Bay (Aomori prefecture).

Water temperature, pH, and salinity at collection time are presented in Table 1. These locations were chosen because migratory birds regularly (every winter) congregate and sojourn in these locations. This means water conditions were not harmful or deleterious for the birds, and, presumably, for the viruses concurrently circulated by the birds.

### 2.2. Viruses

Two low-pathogenic avian influenza viruses, A/Northern pintail/Akita/714/06 H5N2 (Akita/714/06 H5N2) and A/Northern pintail/Aomori/395/04 H7N1 (Aomori/395/04 H7N1) [14], were used for longitudinal observations. The viruses were isolated from fecal materials of migratory, apparently healthy northern pintail (*Anas acuta*) ducks in Japan. The viruses were isolated in embryonated chicken eggs (ECE). Working stocks of viruses were prepared by the third passage in ECE, and allantoic fluid was harvested at 3 days after inoculation (dpi).

### 2.3. Cells Used for Virus Assaying

Human colon adenocarcinoma (Caco-2) cells were shown to well support AIV propagation [15]. Cells were maintained in Dulbecco's Modified Eagle Medium (DMEM, Nissui Pharmaceutical Co. Ltd., Tokyo, Japan), supplemented with penicillin 100 units/mL, streptomycin 100 *μ*g/mL, amphotericin B 0.5 *μ*g/mL, 4 mM L-glutamine, and heat-inactivated 10% (V/V) fetal bovine serum (FBS), and were incubated at humidified environment at 37°C in CO_2_ (5%) incubator. When necessary, cells were maintained in serum-free medium.

### 2.4. Seeding of Water with Viruses

Before virus seeding, collected waters were checked twice in ECE and confirmed as negative for any haemagglutinating viruses. Allantoin fluid containing each of the viruses was diluted (1 : 5) with each of the three types of water and aliquot (500 *μ*L/tube). The waters seeded with viruses (thereafter referred to as sample) were then stored at −20° and −30°C. At each temperature six sets of sample (each virus in 3 types of water) were stored. Besides, allantoic fluids containing each of the two viruses (500 *μ*L/tube) were also stored at −80°C. Samples were taken at predefined interval for titration. 

### 2.5. Preparation of Samples for Titration

For titration, each of the samples from each temperature was taken out and mixed with the equal volume (500 *μ*L for each tube) of phosphate-buffered saline (PBS pH7.2) supplemented with 100x antibiotics [16] and incubated at room temperature for 2 hrs. This gives the virus (virus in sample) dilution 1 : 10. Upon incubation, the sample was transferred into Eppendorf tube and centrifuged at 12000 ×g for 3 min. Allantoic fluid stored at −80°C also incubated at room temperature for 2 hrs.

### 2.6. Titration of Samples

The viruses were titrated monthly in Caco-2 cells supplemented with tosylamide phenylethyl chloromethyl ketone-(TPCK-) treated bovine pancreatic trypsin (Sigma, USA), at the dose rate of 2 *μ*g/mL (final concentration) [17]. Titrations were done using standard protocol. Briefly, a 96-well tissue culture plate (Cellstar, Greiner Bio-one) was seeded with ~7 × 10^4^ cells/well. To remove floating dead cells, at 100% confluent monolayer, cells were washed thrice with PBS (pH7.2) and finally replaced with serum-free medium (100 *μ*L/well) containing trypsin of 4 *μ*g/mL. A 10-fold serial dilution of each of the sample was prepared with 2x serum free medium. After inoculation of cells with diluted samples (100 *μ*L/well, which brings trypsin concentration of 2 *μ*g/mL, and four wells for each dilution), the plate was incubated at 37°C for 3 days. Wells defined as negative control were inoculated with serum free medium. Presence of virus in the culture fluid was determined by haemagglutination test at 3 dpi, and the virus titer, namely, median tissue culture infection dose (log10 TCID_50_), was determined [18]. 

### 2.7. Repeated Freezing and Thawing

Seven months after seeding (which is roughly an average longevity of seasonal lake ice across the Tundra and Taiga), sample (two tubes) containing the H7N1 virus Aomori/395/04 seeded in fresh water and stored at −20°C was taken out and incubated at 10°C for 10 min to facilitate melting. Subsequent to melting, 250 *μ*L of sample from one tube was aliquot and kept on ice. The Rest of the samples (both tubes) were allowed to freeze at −20°C for 75 min. Subsequent to freezing, the sample was taken out again and allowed to melt, aliquot as mentioned, repeatedly up to 4 times. The samples were then prepared as mentioned above, except addition of 250 *μ*L 100x antibiotic instead of 500 *μ*L, and titrated as mentioned.

### 2.8. Profiling Occurrences of Viral Genetic Conservation 

Independently of the above, and in order to fundamentally examine the feasibility of viral genetic conservation taking place due to environmental cryopreservation, we profiled the occurrences of apparent protracted genetic conservation, in terms of literary observational data, with reference being made to a variety of IAV strains at large. “Protracted genetic conservation” is here methodologically regarded as such that represents a mutational rate lower than the minimal ordinary one, compatibly with the following, well-established empirical array. Mutational rate of IAVs has been established, empirically, at 0.001 to 0.007 per nucleotide per year, a variance attributed to different gene segments and different hosts [19–25]. Each gene resembles a biomolecular clock which indicates a regular genetic mutational drift, a delayed drift, or a dormant state. The validity of the biomolecular clock mechanism has been further demonstrated, instantly, for the NS and the HA genes within different host-related strains isolated from humans [26, 27], horses [28], pigs [29], wild ducks [30], domestic ducks [31], and chickens [32]. 

## 3. Results

**Figure 1:**
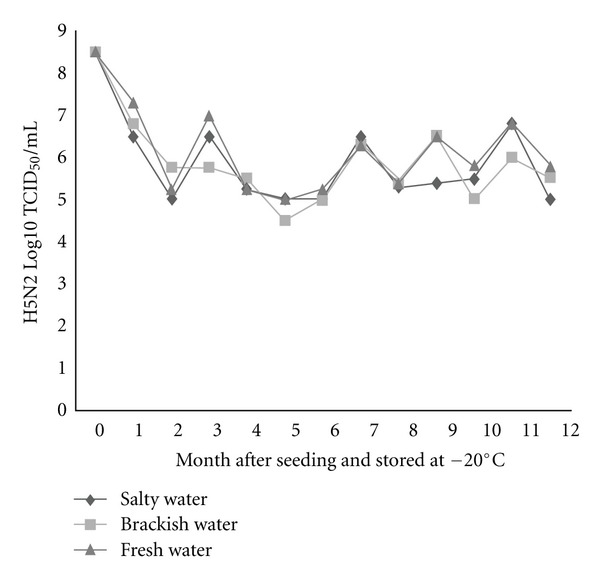
Monthly survival of H5N2 at −20°C in three types of experimentally frozen environmental water.

**Figure 2:**
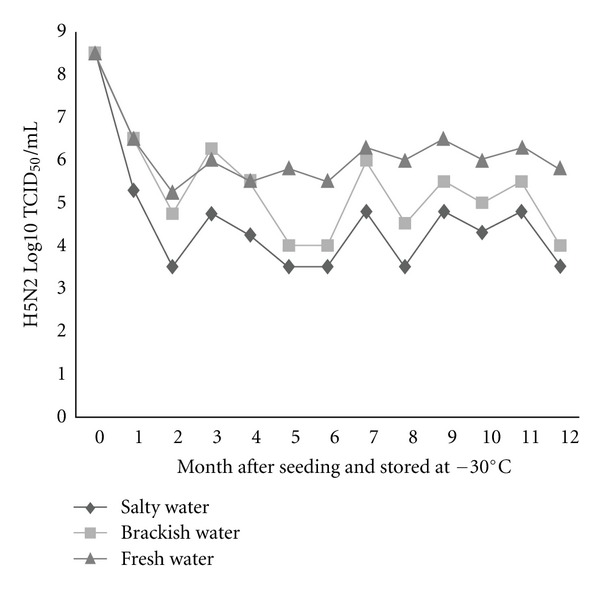
Monthly survival of H5N2 at −30°C in three types of experimentally frozen environmental water.

**Figure 3:**
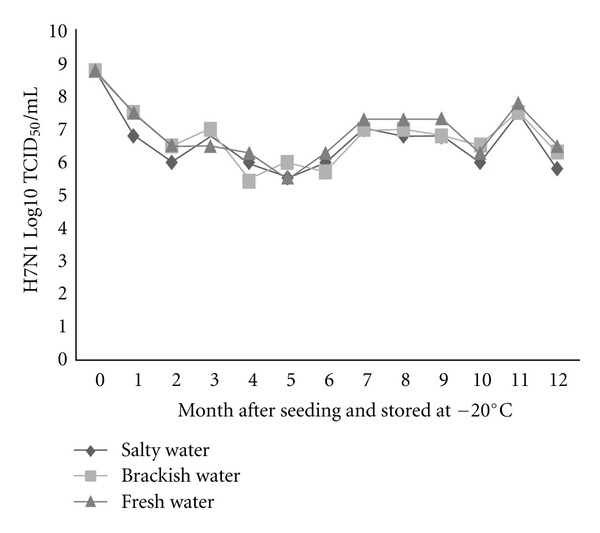
Monthly survival of H7N1 at −20°C in three types of experimentally frozen environmental water.

**Figure 4:**
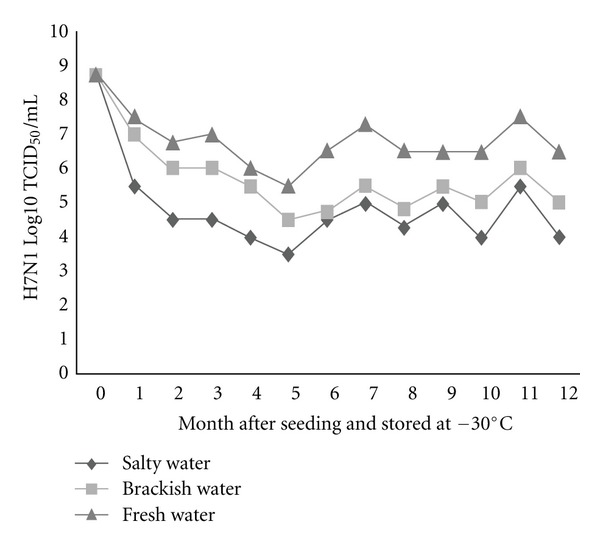
Monthly survival of H7N1 at −30°C in three types of experimentally frozen environmental water.

To substantiate the assumptions that influenza virions can endure freezing and persist viably in the frozen state in nature until thawing occurs, two low-pathogenic H5N2 and H7N1 AIVs were seeded separately in three different types of environmental water (salty, brackish, and fresh water), at the dose rate of 8.5 (H5N2) and 8.75 (H7N1) log10 TCID_50_/mL and stored at −20 and −30°C. Survivability of these viruses was appraised monthly for 12 months in Caco-2 cell line and expressed as log10 TCID_50_. Besides, survivability of one of these two viruses throughout repeated freezing and thawing cycles was also evaluated. All three types of water were confirmed as negative for contamination with AIVs before seeding with the tested viruses. In general, live viruses were detected in all types of water stored at different temperatures at the end of the freezing period, namely, 12 months after seeding. However, the rate of survivability was found to vary among water types, between freezing temperatures, and between virus subtypes. The survivability was found in decreasing trend, meaning, fresh water > brackish water > salty water, irrespective of virus subtype, and storage temperature (Figures 1, 2, 3, and 4). The titer of H5N2 virus stored at −20°C decreased from initial 8.5 to 5.8 log10 TCID_50_ at 12 months after seeding in fresh water, whereas in case of brackish and salty water the titer decreased to 5.5 and 5.0 log10 TCID_50_, respectively (Figure 1). Similar trend was observed with regard to H7N1 virus, irrespective of storage temperature.

The survival rates of H7N1 and H5N2 viruses in freshwater were found to be 74.3%, and 68.2% respectively, regardless of storage temperature. While the survivability of H5N2 virus in brackish water was 64.7% (−20°C) and 47.0% (−30°C), it was appreciably lower in salty water, namely 58.8% (−20°C) and 41.2% (−30°C). Grossly, higher viability of both the viruses was observed during storing at −20°C, as compared with storing at −30°C, except for the viruses seeded in freshwater. About 1.5 and 1.3 Log higher titer of H5N2 and H7N1 viruses, respectively, was found in case of viruses seeded in brackish water stored at −20°C than the viruses seeded in same water but stored at −30°C (Figures 1, 2, 3, and 4). A similar trend was also observed in case of viruses seeded in salty water and stored at −20°C.

In order to ascertain the postulation that influenza viruses can survive repeated freezing and thawing, two tubes of H7N1 viruses seeded in freshwater and stored at −20°C were used at seven months after seeding. Four freezing-thawing cycles were thus monitored. The results of this experiment are shown in Table 2. The cumulative reduction of virus titer at 4th cycle of repeated freezing and thawing was about 25.7% log10 TCID_50_. In other words, the viability of H7N1 virus was about 74.3% log10 TCID_50_. Most of the reduction, 20%, occurred during the first two cycles, reflecting abiotic selection of the majority of cryobiologically unfitted virions.

Observational data regarding protracted genetic conservation taking place within a variety of IAVs, as published in different works, are profiled in Table 3. While the first case presented in Table 3—strain A/pintail duck/Akita/714/2006—points out conservation exhibited by a virus we isolated and analyzed, all the other cases presented in that table are mentioned elsewhere, as referenced. The data presented in Table 3 reflect an array including considerable durations of viral-protracted genetic conservation, from as little as 3 to as much as 82 years, and pertain to examples of avian, porcine, and human viral strains, isolated throughout 1977 to 2006.

## 4. Discussion

The main purposes of this study are (1) to conceptualize that AIVs are capable to and probably do undergo and survive frozen state in various types of environmental water, which are naturally and regularly seeded with AIVs, while occupied by aquatic bird and thereby (2) to better comprehend the entire life cycle and evolution of AIVs, with its broad implications. Beyond the biophysical feasibility of such cryotolerance, the related components of ecological feasibility and genetic feasibility are vital, too, in that they are altogether interlinked and would hence conjunctively substantiate the paradigm posed in the present study. Therefore, those three feasibilities are herewith discussed in details, and each of them pertains to the characteristics and results of the present study.

### 4.1. Ecological Feasibility

Biogeographically, the overall system of aquatic bird pathways forms a global mosaic that interconnects in effect any water body worldwide through the migration routes of innumerable species. As a result, throughout any whole given year (starting in spring), any current or ice-released AIV strain can in principle be conveyed between any two watery loci on Earth, by means of one avian host species, or, consecutively, more than one species. Therefore, in the long run, there exists a constant cycling of the biotic and abiotic pools of viruses. 

Large portions of the entire cryosphere may be regarded and explored as chief IAV abiotic reservoir and supplier. The related viral inventory is hence fully conveyable, conceivably, thanks to the permanent presence of many influenza virus-permissive waterfowl, shorebirds, seabirds, and marine mammals (seals and whales) up to the northerly marginal ice zone. Moreover, they are mostly nomadic and therefore are readily capable of acting as effective disseminators of the virus strains.

Inland, dabbling ducks reach most northern lakes and ponds via seasonal migration during summer, following the northerly progressing line of ice melting, until the end of August. Marine environments are less studied. Seagulls commonly host AIV and serve as origin of strains that infect seals and whales. Additional seabirds (Alcidae, at the rate of infectedness of 1.4%), and sea ducks (Anatidae, tribe Mergini, 0.7%), are known to host AIVs [45]. Common and king eider sea ducks (*Somateria mollissima* and *S. spectabilis*, resp.) are prevalent throughout the Arctic Ocean islands and coasts, even during wintertime, yet they associate with dabbling ducks across the arctic tundra lakes during summer, while breeding, and are thus exposed to freshwater viral strains, as well. Viral diversity and circulation are amplified by seals and whales. All in all, arctic sea water is continuously seeded with avian and mammalian influenza viruses. While immense virus dilution is caused by sea water, virus survival in the Arctic Ocean might be supported by very low water temperature and long-lasting ice.

In Antarctica, AIVs were detected only serologically (in penguins [46]), but the Antarctic cryosphere should not be excluded as having, to a certain degree, a cryobiological role which is similar to the northern cryosphere, regarding the ecology of IAV. Antarctica, albeit the paucity of waterfowl found in it (only the southern pintail *Anas eatoni* and speckled teal *A. flavirostris*), is frequented by multiple seabirds—part of which hosts AIVs, potentially—and comprises (in difference of the Arctic Ocean) a huge land with many freezing-thawing freshwater bodies. Also, an interface between holarctic and antarctic aquatic birds exists and presumably introduces multiple AIVs into southern avifaunas. The arctic tern (*Sterna paradisaea*), a species known to host AIVs and migrate from pole to pole [47], conspicuously illustrates this apparatus.

In the present study, we used ordinary AIV strains isolated from typical migratory ducks that prevalently occupy ponds and lakes in Canada and Siberia each year from about April to October, thus following the ecological paradigm mentioned above. We therefore used surface water from freshwater lake and brackish water lake that are seasonally resided in by pintails (and other migratory ducks), as well as sea water, for comparison.

### 4.2. Biophysical Feasibility

Lake ice in the sub-arctic [48] and sea ice in the Arctic [49, 50], as well as lake ice [51] and sea ice [52] in Antarctica, were found to be an advantageous natural preserver for viruses that are hosted by multiple cold-adapted aquatic bacteria and algae species. Viruses and bacteriophage (as prophage) frozen in glaciers can be preserved for over 100,000 years [53]. As for influenza virus, the thermal amplitude is much more complex, since the virus has to be adaptable to body temperatures of mammals and birds—meaning from 37°C to 41°C—at the same time. A variety of RNA and DNA viruses hosted by homoeothermic species, including influenza viruses, were found to withstand experimental freezing, storing, and thawing—even cyclically (refrozen)—to appreciable or full degrees [54]. Specifically, the recovery rate of influenza A viruses, subsequent to experimental prolonged freezing and thawing, was shown to be marked [55, 56]. However, the viruses tested were human strains, whereas avian strains presumably constitute the natural, primarily ice-adaptable strains. 

Virus survival in environmental water is a prerequisite for subsequent frozen phase, and endurance in freshwater for months has indeed been evidenced. It was found that AIVs persist viably in freshwater, brackish water, and sea water, in converse correlation with temperature and salinity, for up to several months, usually [57–59]. Considerably extended persistence, up to 490 days, was estimated for an ordinary avian influenza strain (H2N4) isolated from a teal, in water at temperature 4°C, pH 7.2, and salinity 0 ppt [60, 61]. Fairly similar conditions prevail in ponds and lakes during summertime across the Taiga and the Tundra.

One study monitored viable persistence of AIVs in experimentally frozen lake water, at −10°C. Viruses isolated from waterfowl (mallard, teal, and swan) were thus found to viably persist for 182 days (starting virus titer of 104.14/mL); 182 days (starting virus titer of 104.5/mL); 224 days (starting virus titer of 105.14/mL) [62]. In Siberia, IAV RNA was found to be preserved in higher concentrations in lake ice than in lake water [63]. In Alaska, AIVs were readily detected during wintertime in sediments found in frozen ponds. Although virus viability was not assayed in the latter study, it was observed that these sediments could constitute a year-to-year reservoir of viruses, which serve to infect birds occupying the ponds [64]. 

Notably, variation in cryotolerance and thermostability of influenza virus isolates begins at the level of virus population. While a given propagating population continuously shapes into more adaptable sub-populations through ongoing genetic changes, a given population undergoing an abiotic liquid or frozen phase cannot alter genetically during such phase. The latter population is therefore merely passively selected, while certain given sub-populations of it-which are more survivable in water or ice—can endure. The presence of AIV sub-populations with high thermostability has indeed been pointed at, suggesting that avian viruses can persist in water longer than previously estimated [65]. Presumably, the same principle is valid regarding cryotolerance.

Biophysically, the frozen phase is rather complex, since it actually includes three stages—freezing, frozen state, and thawing—each coped with by the virus. Viruses with lipid envelopes, such as influenza, are often less stable than nonenveloped viruses at ambient temperatures, but survive well at ultralow temperatures [66]. It has been pointed out, indeed, that avian influenza viruses might survive indefinitely when frozen in the environment [67, 68]. Specifically, it has been observed that the envelope of IAVs in general (as demonstrated in the strain ×31 Japan) is remarkably and uniquely stable with freezing and thawing, gradually solidifying from an oily fluid into a hardened gel, without sudden changes, while temperature is decreasing [69]. 

Further, by electron cryomicroscopy, it was shown that the human IAV strain PR/8/34 in frozen state has two different sub-populations, one exhibiting high viability and composed of filamentous and small spherical virions, and another one exhibiting low viability and composed of large spherical virions. The envelopes of most of the filamentous and small spherical, viable virus particles showed a combination of a thin—hardened gel, probably—lipid monolayer (plus a thick protein-containing inner layer), while the envelopes of most of the large, nonviable virus particles comprised phospholipid bilayers (that did not harden, apparently) [70]. 

In the present study, the main results presented are of the effect of storage in ice at −20°C and −30°C for 1–12 months. As expected, there is no much difference between 1 month and 12 months, much of the loss of viability taking place during the freezing and/or thawing process, with the duration of storage frozen having minimal effect. Biotic virus recirculation is much dependent, though, upon the viability of virus after thawing. Birds may not get infected immediately following the thawing of the ice, and whether the virus remains viable in close-to-freezing temperatures for many days in such waters is a vital property, then. AIVs are indeed prone to have that property particularly that the postthawing virus population is a derivative of the water-enduring prefreezing virus population. The findings are compatible with the proposed natural cryopreservation apparatus. We thereby showed that two ordinary avian strains—H5N2 and H7N1—isolated from a typically widespread migratory duck (northern pintail) proved appreciably stable during 12 months of frozen phase, with its three stages. It can be deduced that natural cryopreservation in perennial ice would be as supportive.

Moreover, we assayed one of the two avian strains that survived for 7 months at −20°C for further survivability under repeated freezing-thawing cycles, and it exhibited considerable endurance, which means, inferentially, that virions released from melting environmental ice and not contracted thereafter by an avian host until refreezing occurs would likely undergo further frozen phase, extending their endurance into another year.

It should be noted that under natural conditions cryotolerance of IAVs that have been excreted in feces by waterfowl into prefreezing lake water or onto lake ice may expectedly be increased, because they are aggregated and relatively stabilized and protected by fecal material. Thus aggregated, they retain, as well, high concentrations [71]. 

In conclusion, the more northerly a given aquatic biotope is located, the greater is the feasibility of the described cryobiological apparatus, because:

 temperature of liquid water is closer to 0°C, thus lengthening survival of virions found in it;
 average temperature of ice is lower, hence more supportive;
 meteorologically, sunlight and UV radiation are further reduced during the period of frozen state (roughly from September to May) and viruses are thus less damaged;
 optically, ice diminishes the penetration of UV radiation (in comparison to liquid water):
 larger portion of water freezes annually;
 larger portion of frozen water remains in the form of perennial ice.

By contrast, in southern sub-arctic lakes, where the liquid phase is prolonged, viral dependence on recovery through a biotic sub-phase might be critical.

Besides, the parameters of salinity and sub-zero temperature are influential, both separately and in conjunction. In liquid water, the persistence of H5 and H7 AIVs was found inversely proportional to temperature and salinity, while significant interaction exists between the effects of temperature and salinity on the persistence, in that the effect of salinity is more prominent at lower temperatures [72]. Our findings, in ice, demonstrate that while no difference in survivability due to varying salinity was observed in −20°C, markedly lower survivability was observed in −30°C as salinity was higher. The water cryodynamics property causing this variance is not clear, but is nevertheless significant since transitions from −20°C to −30°C are quite often in the Arctic and sub Arctic.

### 4.3. Genetic Feasibility and Protracted Gene and Genome Conservation

Table 3 demonstrates that protracted genetic conservation occurs both at the level of gene segments and whole genomes. Indeed, due to the commonness of viral gene reassortments marking IAV, practically any IAV gene segment (though not any genome), whether mammalian- or avian-derived, can tentatively be contracted by migratory aquatic birds, and thereby disseminated and perpetuated worldwide [73]. As a result, virtually any viral gene (though not any genome) of IAV can undergo preservation in annual or perennial environmental ice, thereafter reappearing genetically conserved to an extent that cannot be accounted for by ongoing regular mutational clock. Since genetic reassortments may occur immediately after ice thawing and resultant recontraction of ice-released viruses by aquatic birds, single-gene segments showing such protracted genetic conservation are as representative of preservation in ice as entire genomes (with which they were necessarily affiliated at some point in the past).

The present study highlights the hypothesis that authentic (namely, not artificially-produced, either knowingly or accidentally) protracted genetic conservation revealed in IAVs is mostly an outcome of preservation in environmental ice, considering that IAVs do not prevail in the form of latent infection. Empirically, protracted genetic conservation has repeatedly been observed in IAVs, both avian and mammalian (Table 3), and is significantly in support of the genetic feasibility of the mentioned hypothesis. For instance, the Eurasian porcine-originated NA and M genes of the recent pandemic H1N1 2009 strain exhibited protracted genetic conservation, alongside with whole genome-protracted conservation (around 20 years at least) recognized within 2% of various swine strains [74]. 

AIVs isolated by us during 4 years from pintail ducks wintering in Japan—including the two strains exhibiting marked cryotolerance in the present study—show persisting genotypic interhomology, which may result from virus preservation in lake ice in the ducks' Siberian breeding grounds [75]. Further, we found that the NS gene of one of those isolates (H5N2) has 98.3% nucleotide homology with a H3N8 virus isolated from duck in 1972 in Chabarovsk, a Russian region lying aside the principal Siberian route of migratory waterfowl. [76] Arithmetically, this means that for at least 11 years, the NS gene has been conserved, considering the ordinary minimal yearly mutational rate (Table 3).

A fairly comprehensive genomic study by Hayashida et al. led them—according to their findings, as detailed in Table 3—to the general conclusion that “frozen replication is not a rare evolutionary event, but is reasonably expectable in influenza virus evolution” [34]. In practice, any duration of a genetic conservation (nonreplicative) phase can be attributed to preservation in environmental ice (particularly that global temperatures heighten). For instance, if a gene of an avian strain isolated in a certain year exhibits maximal nucleotide sequence homology to a gene of an avian strain isolated 30 years earlier, while the degree of homology arithmetically reflects mutational stasis of 10 years, it means that this gene was preserved in environmental ice for 10 years in total, either continuously or discontinuously, during those 30 years. Basically, the genetic conservation phase may last for thousands of years, and one can assume that such a frozen genetic inventory includes certain portions indicating the historical genomic evolution of IAVs, whether in the form of genetic material or viable virions.

Worobey strongly depreciated the possibility that evolutionary stasis—whether due to preservation in ice, or otherwise—takes place in effect within IAV [77]. Contrastingly, however, Webster observed that influenza viruses perpetuated within aquatic bird populations do undergo evolutionary stasis, for already decades (to the least) [78]. This lasting equilibrium is interrupted only by the contraction of different strains harbored by nonaquatic birds [79]. At any rate, instead of primarily examining the actual feasibility, or evidencing infeasibility, of the proposed virus preservation in environmental ice, Worobey principally discredits such mechanism and concentrates, resultantly, on trying to repeatedly prove laboratory artifacts, contamination, outwards leakages, and alike—which are potentially important misleading factors, certainly, hence can endlessly serve for plausible argumentation, ostensibly—as true explanations for each and every finding of protracted gene/genome conservation. Thus, well-established phylogenetic paradigms of fully continuous evolution are used in order to negate any findings of such conservation, while in reality such findings are of course not intended to constitute an alternative paradigm, but a complementary one. Rather more objectively, findings of protracted influenza gene or genome conservations were elsewhere referred to as such that “may point to surprising new biology but are perhaps more readily explained by stock contamination or other errors in the sequencing laboratories” [13]. The issue, however, is not one of new biology, but an approach elucidating unusual findings through a feasible, mostly unnoticed natural mechanism—which is not supposed to replace existing concepts—rather than just considering such findings to be artifacts. Anyhow, it should be emphasized that although at least some, rather than none, of the various controversial findings of protracted genetic conservation can be regarded as reliable, they constitute at any rate but a secondary rationale for the present study. The primary and adequate rationale is the plain challenge to explore the feasibility-which is remarkably meaningful, potentially-that influenza virions commonly found in environmental freshwater (and, apparently, seawater) upon freezing are consequently preserved viably in the frozen state and thereafter released infectious from the melting ice. In case some of the protracted genetic conservation findings are valid, then they are significantly supportive of such feasibility, whereas even if none is valid, which is fairly improbable, this would not at all depreciate the cryobiological apparatus proposed here. In practical terms, a sound body of observational data and experimental findings obtained in our study and elsewhere, as detailed, altogether readily substantiates the cryobiological concept posed in the present study, and the related ecological, biophysical, and genetic feasibilities too are well compatible with that concept, as demonstrated.

Temporally, abiotic virus preservation in annual (seasonal) ice may consume 33% of the time (4 out of 12 months per year, in the southern Tundra), and up to 83% (10 out of 12 months per year, in the northern Tundra). In accordance, during a decade, for instance, utmost discontinuous preservation taking place in such manner (not necessarily in the same lake) would accumulatively bring about genetic conservation of 40 to 100 months, respectively.

Genetically, since influenza gene pool harbored by avian hosts includes human and porcine genes [80–82], the proposed cryobiological apparatus principally allows for such genes as well to undergo the same course and thereafter be contracted by aquatic birds and conveyed onto poultry and pig farms. Intact avian influenza genomes too are most probably prone to resurface and recirculate in that fashion. Perennial preservation in ice may basically last for few up to thousands of years and can thus significantly affect evolutionary, epizootical, and pandemic mechanisms.

## Figures and Tables

**Table 1:** Source and properties of different types of water seeded experimentally with the viruses.

Type of water	Source	Collection date	Temp (°C)	pH	Salinity (%)*
Salty	Mutsu Bay Hiranai, Aomori	December 17, 2008	14.9	7.93	0.9
Brackish	Lake Ogawara, Aomori	December 17, 2008	14.1	8.4	<0.2
Fresh	Lake Izunuma, Miyagi	December 20, 2008	19.8	7.1	<0.2

*Detection limit was 0.2%.

**Table 2:** Cumulative effect of experimental successive freezing-thawing cycles on AIV survival in lake water at −20°C (strain H7N1 A/northern pintail/Aomori/395/04).

Freezing and thawing cycle^a^	Virus titer TCID_50_/mL (Log10)*	Cumulative reduction (% TCID_50_)
First	7.75	11.4
Second	7	20.0
Third	6.75	22.0
Fourth	6.5	25.7

^a^Freezing conducted at −20°C for 75 minutes and thawing at +10°C for 10 minutes.

*Initial titer upon seeding was 8.75 log10 TCID_50_/mL.

**Table 3:** Examples of protracted gene and genome conservation in IAVs.

Genes	Late isolate	Subtype	Duration of conservation	Early isolate	Subtype
NS	A/pintail duck /Akita/714/2006	H5N2	11/34 (11 out of 34 years)	A/duck/Chabarovsk/1610/1972	H3N8 [33]
NA	A/duck/Ontario/16/1977	H2N1	9/27	A/FW/1950 (human)	H1N1 [34]
NA	A/USSR/90/1977 (human)	H1N1	25/27	A/FW/1950 (human)	H1N1 [34]
All except M	A/USSR/90/1977 (human)	H1N1	≤27	A/FW/1950 (human)	H1N1 [35]
M	A/USSR/90/1977 (human)	H1N1	≤30	A/FM/1947 (human)	H1N1 [35]
All	A/Mongolia/111/ 1991 (human)	H1N1	≤57	A/PR/8/1934 (human)	H1N1 [36]
HA	A/Alma Ata/175/ 1983 (human)	H1N1	≤53	A/swine/Iowa/15/1930	H1N1 [37]
HA	A/lake Park ice/ Siberia/2002	H1N1	≤64	A/swine/UK/1938	H1N1 [38]
NP and HA	A/pintail duck /Ohio/25/1999	H1N1	≤82	A/Brant Goose/ 1/Alaska/1917	H1N? [39]
All	A/duck/Vietnam/568/2005	H5N1	≤8	A/chicken/Hong Kong/220/1997	H5N1 [40]
PB2	A/chicken/Taiwan /G23/1987	H6N1	15/15	A/duck/Taiwan /526/1972	H6N1 [13]
HA	A/ChaingMai/4/1985 (human)	H3N2	3/3	A/Philippines/2 /1982 (human)	H3N2 [41]
HA and NP	A/swine/Quebec/ 296/1994	H3N2	≤19	H3N2/Canada/1975 (human)	H3N2 [42]
HA and NP	A/swine/Quebec/ 148/1990	H1N1	≤60	A/swine/Iowa /15/1930	H1N1 [43]
All	A/Baku/799/1982 (human)	H1N3	≤6	A/whale/Pacific Ocean/19/1976	H1N3 [44]
